# Affine Transform to Reform Pixel Coordinates of EOG Signals for Controlling Robot Manipulators Using Gaze Motions

**DOI:** 10.3390/s140610107

**Published:** 2014-06-10

**Authors:** Muhammad Ilhamdi Rusydi, Minoru Sasaki, Satoshi Ito

**Affiliations:** 1 Department of Mechanical Engineering, Gifu University, 1-1 Yanagido, Gifu City, 501-1193, Japan; E-Mails: q3812204@edu.gifu-u.ac.jp (M.I.R.); satoshi@edu.gifu-u.ac.jp (S.I.); 2 Department of Electrical Engineering, Andalas University, Limau Manis, Padang City, 25163, Indonesia

**Keywords:** EOG, gaze motions, affine transform, linear relationship, actual pixels, target pixels, robot manipulator

## Abstract

Biosignals will play an important role in building communication between machines and humans. One of the types of biosignals that is widely used in neuroscience are electrooculography (EOG) signals. An EOG has a linear relationship with eye movement displacement. Experiments were performed to construct a gaze motion tracking method indicated by robot manipulator movements. Three operators looked at 24 target points displayed on a monitor that was 40 cm in front of them. Two channels (Ch1 and Ch2) produced EOG signals for every single eye movement. These signals were converted to pixel units by using the linear relationship between EOG signals and gaze motion distances. The conversion outcomes were actual pixel locations. An affine transform method is proposed to determine the shift of actual pixels to target pixels. This method consisted of sequences of five geometry processes, which are translation-1, rotation, translation-2, shear and dilatation. The accuracy was approximately 0.86° ± 0.67° in the horizontal direction and 0.54° ± 0.34° in the vertical. This system successfully tracked the gaze motions not only in direction, but also in distance. Using this system, three operators could operate a robot manipulator to point at some targets. This result shows that the method is reliable in building communication between humans and machines using EOGs.

## 1. Introduction

The electrooculography (EOG) signal is a bio-signal that measures eye activities. Those activities generate a potential difference between the cornea and the retina. Although many methods can learn the phenomena of eye movement, the potential difference [1] is the most broadly used by neuroscientists to investigate eye movements [2]. The EOG signal has a linear characteristic with regard to the gaze motion distance [3]. This linear condition of the EOG happens at approximately ±45° in the horizontal direction and ±30° in the vertical [4].

Some methods have been developed to determine the relationship between the EOG and gaze motions. Linear predictive coding cepstrum (LPPC) was used by [5] as the feature for eye movement pattern matching. In various experiments, this research also used dynamic time warping to compensate the EOG period. A spectral entropy algorithm was implemented to detect the endpoints of the EOG to improve the accuracy of the recognition system among noisy signal conditions. The purpose of the experiments in this study was to detect seven eye activities when looking up, down, left, right, blinking two times, blinking three times and blinking four times.

Barea *et al.* [6] proposed a system to identify horizontal gaze motions based on a neural network. They obtained ±10° with a 2° error accuracy for horizontal gaze motion. A neural network for EOG signal recognition was also implemented to help physicians make a diagnosis to distinguish normal and subnormal eye conditions [7]. A neural network for classifying the EOG in six conditions was also performed by [8]. The conditions that were distinguished were straight, up, down, right, left and blink. Combinations between three EOG features, which were the wavelet detail coefficient, power spectral density and auto regressive coefficient, were also performed to compare their accuracy.

Fuzzy logic was implemented for detecting eye movement by using the EOG in [9]. The horizontal EOG was divided into four groups: right, left, hard right and hard left. The vertical EOG was grouped into up and down only.

A low-cost computer interface based on the EOG was proposed by [10]. This interface used the polarity of signals to differentiate the gaze motion direction on each channel. The peak amplitude and the slope of the signals were used to categorize the blink, eye movement and noise. This method successfully recognized right, left, up and down gaze motion and blinks.

In [11], the integral value of the EOG was introduced as a feature to detect eye movement. Two interface models were controlled by three operators. The first had eight options and the other had 12 options for gaze motion. This method showed better accuracy for both models as compared to the maximum EOG value method.

Several researchers have already invented some applications. Pinhero *et al.* [12] reviewed EOG functions to help disabled people communicate with other people through a machine controlled by EOG. EOG signals were converted into alphanumeric/symbol/number and cursor control signals and also used to generate Morse code. EOG was also used to operate a TV and play games [13].

This paper proposes an affine transform method to build a gaze motion tracking system. A homogeneous affine transform was constructed by sequences of five geometry processes, which are translation-1, rotation, translation-2, shear and dilation. This method was designed to detect the direction and the distance of gaze motions. Three operators tested the system by gazing at 24 target points. Finally, a robot manipulator was used as the indicator of gaze motions to some target points.

## 2. Experimental Environment

### 2.1. EOG Signal

**Figure 1.**
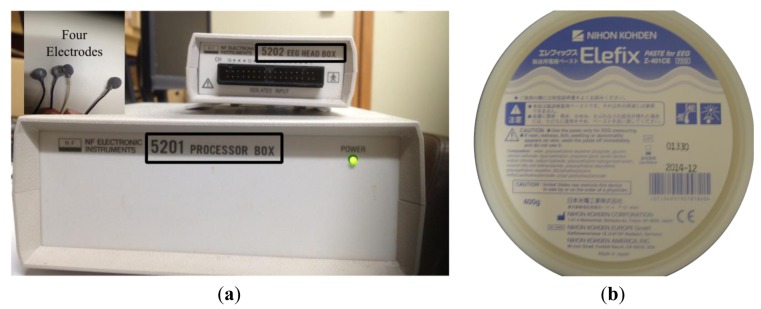
(**a**) NF instrument for EOG sensors with a processor box, a head box and four electrodes. (**b**) Elefix paste produced by Nihon Koden.

This research focused on building an eye tracking system of direction and distance of gaze motions using EOG signals. An instrument produced by the NF Corporation (Yokohama, Japan) was used to measure the EOG. This sensor has four electrodes, a processor box and a head box as the system amplifier, as shown in Figure 1a. A 60 Hz low-pass filter, as shown by Equation (1), was used inside the processor box, since standard electric noise occurred even when the electrodes were not attached on the skin. Elefix paste (Nihon Koden, city, country), a highly conductive and low impedance gel as shown by Figure 1b, stuck the electrodes to the dry skin, so the electrodes were in stable positions:

(1) $$\begin{array}{c} \text{H}\left(\text{s}\right)=\frac{4\pi^{2}f^{2}}{s^{2}+4\pi \text{fcos}\left(\frac{\pi }{4}\right)+4\pi^{2}f^{2}}\\ f=60\text{Hz cutoff frequency} \end{array}$$

**Figure 2.**
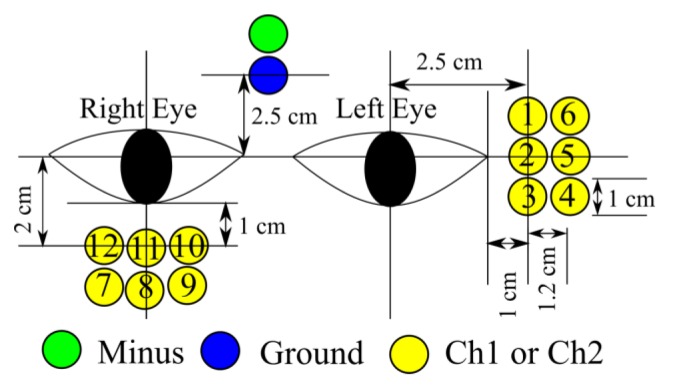
Possibilities of electrode positions.

The electrodes consisted of a ground channel, a reference channel, channel 1 (Ch1) and channel 2 (Ch2). Ch1 detected the signal for vertical gaze motions and Ch2 for horizontal gaze motions. EOG is very sensitive to electrode position and many different methods to record the EOG signal are used in different laboratories [14]. In [15], some electrode position possibilities, as shown by Figure 2, were checked based on average and standard deviation of the integral EOG for some gaze motion distances. The result showed that electrode position number 2 for horizontal gaze motions (Ch2) and number 11 (Ch1) for vertical gaze motions had a stable EOG signal and linear relationship with gaze distance.

Three features of the EOG signal were used in this study. First were the threshold values. These were types: the positive threshold (Th+) and the negative threshold (Th−). The signals between these thresholds indicated condition of eyes (at rest/no movement). Second was the polarity of the signal from Ch1 and Ch2. The polarity was defined from which threshold (Th+ or Th−) was passed first by the EOG. Positive signal (+) was the condition for EOG signal if it first passed the positive threshold and negative polarity was the condition for EOG signal if it first passed the negative threshold.

If the polarity of the signal of Ch1 was negative (−) and the polarity of the signal of Ch2 was negative (−), they were grouped into Area 1. In Area 2, the polarity of the signal of Ch1 was positive (+) and the polarity of the signal of Ch2 was negative (−). In Area 3, both of the polarities were positive (+). In Area 4, the polarity of the signal of Ch1 was positive (+) and the polarity of the signal of Ch2 was negative (−). Figure 2 illustrates these conditions and Table 1 lists them.

**Figure 3.**
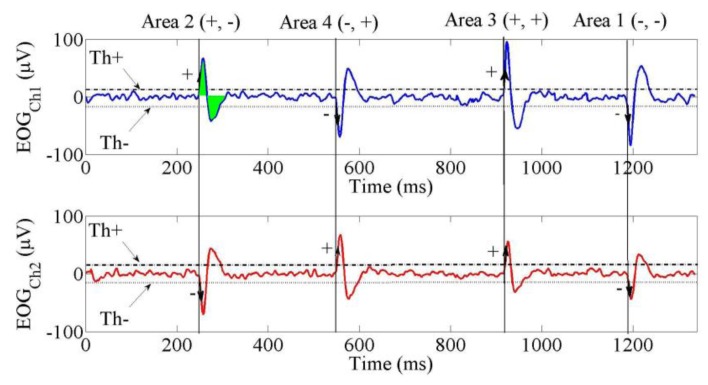
EOG signals for Areas 1, 2, 3 and 4.

The last EOG feature in this experiment was its integral, which had a linear relationship with gaze distance. In Figure 3, the green shadow for the EOG signal from Ch1 in Area 2 shows an example of integral of EOG. The integral was calculated from the first time the signal passed the zero value until it constructed a full wave, as shown by Equation (2). The integral of EOG was normalized to scale the data into 0 to 1. The linear relationship between normalized of integral EOG and eye distance is shown by Equation (3):

(2) $$\begin{array}{cc} \text{int\_}{\text{EOG}}_{\text{Chi}} & =|\int_{\Omega_{+}}{\text{EOG}}_{\text{Chi}}\left(t\right)\text{dt}|+\left|\int_{\Omega_{-}}{\text{EOG}}_{\text{Chi}}\left(t\right)\text{dt}\right|\\ \Omega_{+} & =\left\{t:{\text{EOG}}_{\text{Chi}}\left(t\right)>\text{th}+\right\}\\ \Omega_{-} & =\left\{t:{\text{EOG}}_{\text{Chi}}\left(t\right)<\text{th}-\right\}\\ i & =1,2 \end{array}$$

(3) $$\begin{array}{cc} \text{Distance} & =\text{A}*\text{norm\_int\_}{\text{EOG}}_{\text{Chi}}+\text{B}\\ \text{i} & =1\;\text{for up or down}\left(\text{v}\right)\text{and}\;2\;\text{for right or left gazemotion}\left(\text{u}\right)\\ \text{Distance} & =\text{v pixel for up or down and u pixel for right or left gazemotion}\\ \text{norm\_int\_}{\text{EOG}}_{\text{Chi}} & =\text{normalization of integral EOG} \end{array}$$

**Figure 4.**
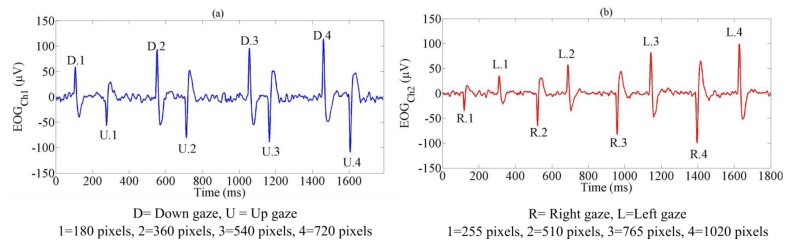
Four kind of gaze motion distances (**a**) EOG signal from Ch1 in vertical direction, and (**b**) EOG signal from Ch2 in horizontal direction.

In the previous research [16], some horizontal and vertical gaze distances were measured to determine variables *A* and *B*. Some horizontal and vertical targets were put on a monitor which was 40 cm in front of operators. The size of the monitor was 34 cm × 27 cm (width × height). The distance between two targets in horizontal was 255 pixels and the distance between two targets in the vertical was 180 pixels. The average values of *A* and *B* for the four basic directions (up, down, right and left) of gaze motion were 900 and −300 for up, −850 and 200 for down, 1,100 and −138 for right and −1,100 and 130 for left gaze motion. The vertical gaze motions (up and down) were associated with movement in *v* pixel and the horizontal gaze motions were organized into the *u* pixel. Figure 4a shows the signal from Ch1 for four gaze distances (180, 360, 540, and 720 pixels) in vertical gaze motions (down and up) and Figure 4b illustrates the signal from Ch2 for four gaze distances (255, 510, 765, and 1,020 pixels) in horizontal gaze motions (right and left).

### 2.2. Integrated System

**Figure 5.**
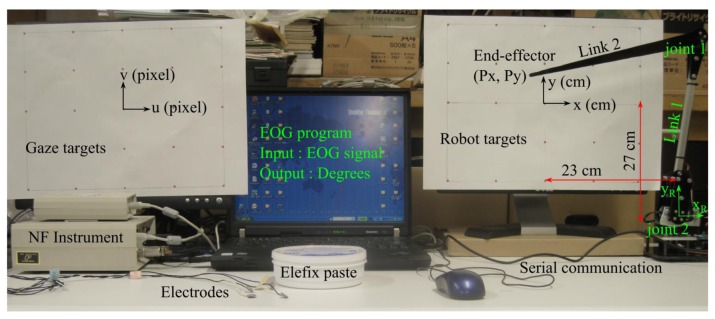
Experiment setup of robot control system using EOG.

Figure 5 illustrates the hardware setup of this experiment. There were two target plates, one is for gaze targets and another is for the robot targets. An EOG program worked with the input was EOG signals and the output was the angle degrees for the robot manipulator.

There were three system coordinates in this research, target coordinate (u,v) in pixel, target coordinate (x,y) in cm and robot coordinate (x_R_,y_R_) in cm. Because of the offside positions between (x,y) and (x_R_,y_R_), the relationship between (x,y) and (P_x_,P_y_) was calculated by Equation (4):

(4) (Px,Py)=(x−23cm,y+27cm)

A planar robot manipulator was used as indicator of eye movements. It had two joints with length of both joints was same, 30 cm. Angle of joint 1 was represented by α and angle of joint 2 was named by *β*. The area of *α* was from 0° to 180° and the area of *β* was from 0° to 140°. The relationship between end-effector position (P_x_,P_y_) and the joint angles are given by Equations (A1)–(A7) in Appendix A. This robot was connected using serial communication between an Arduino microcontroller and a computer. This computer received EOG data from the processor box.

## 3. Methodology

Three human operators attempted to track their gaze motion by using EOG in this research. Their heads were fixed 40 cm in front of a monitor, where the target points appeared. A sequence of experiments was conducted to find the gaze motion pixel coordinates or actual pixel positions. A pixel coordinate was evaluated by comparing it to the target pixel position. The geometry process was used to improve the performance of the gaze motion tracking system.

### 3.1. Training Targets

**Figure 6.**
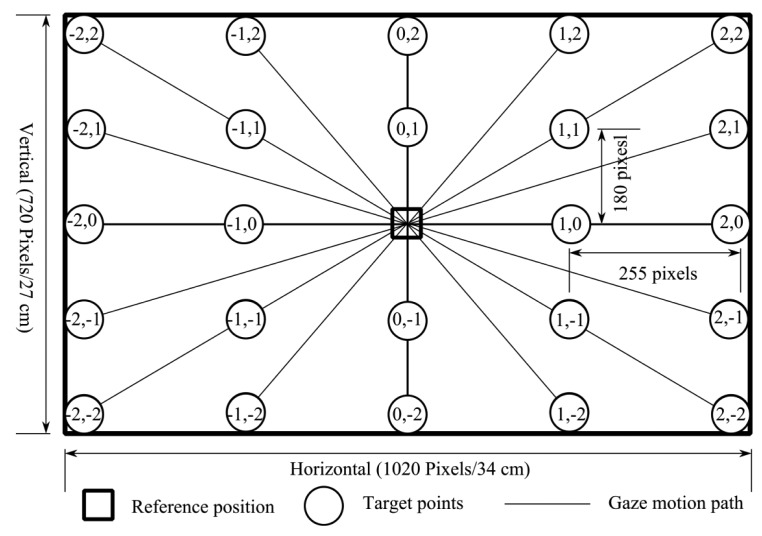
Training targets and eye reference positions.

In total, 24 training targets used in this research were symmetrically spread across the monitor, as shown in Figure 6. The size of the monitor was 1,020 pixels × 720 pixels or 34 cm × 27 cm (horizontal × vertical). Every pixel in the horizontal and vertical could be converted to cm unit by Equation (5). The monitor was 40 cm in front of operators. Using trigonometry rules, the 255 pixels in horizontal directions were equal to 12° and 180 pixels in vertical directions was approximately 9°. The training targets were named based on their location. Table 2 lists the target names and their pixel positions. The areas of the targets were based on the EOG signal type:

(5) $$\left(x,y\right)=\left(\frac{u*34}{1020}\text{cm},\frac{v*27}{720}\text{cm}\right)$$

The three operators performed gaze motions from the reference position to the target points. A reference point was the (0,0). Each movement was done five times for a total of 120 gaze motions.

### 3.2. Reference Line

**Figure 7.**
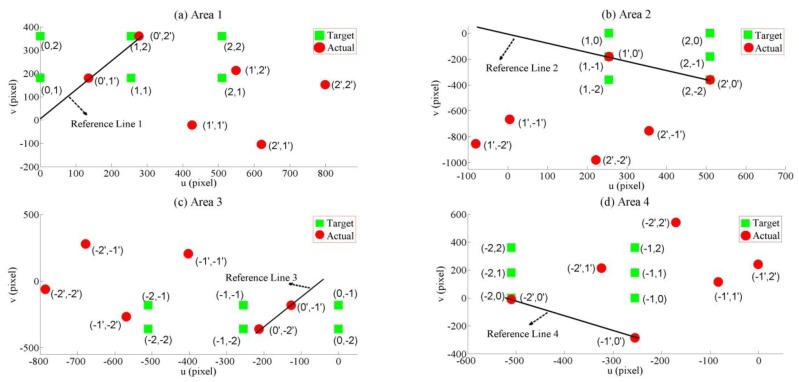
Actual and target pixels from the EOGs.

Figure 7 shows the patterns of the actual pixel positions in the four areas. The green squares are the target pixel positions. The red circles are the actual pixels from Ch1 and Ch2. Each area had six target positions and six actual positions.

Each area had a reference line. A reference line was needed since the rotation geometry process is the projection of the actual pixels to this line. In addition, these lines were also necessary to perform the shear geometry process. At a glance, the actual pixels could be grouped into two patterns. Area 1 had reference line 1, which was a linear line drawn between the actual pixels at (0′,1′) and (0′,2′). Area 3 had the same pattern as area 1, but reference line 2 was drawn between the actual pixels at (0′,−1) and (0,−2). Area 2 and 4 drew their reference lines from actual pixels that occurred from the horizontal gaze motion. In this case, reference line 2 was drawn between the actual pixels at (1′,0′) and (2′,0′), whereas the actual pixels at (−1′,0′) and (−2′,0′) composed reference line 4. Since the reference lines are linear, they are simply written by Equation (6):

(6) $$y=a_{i}x+b_{i},i=\text{area}\left(1;2;3;or4\right).$$

### 3.3. Homogeneous Matrix of the Affine Transform

**Figure 8.**
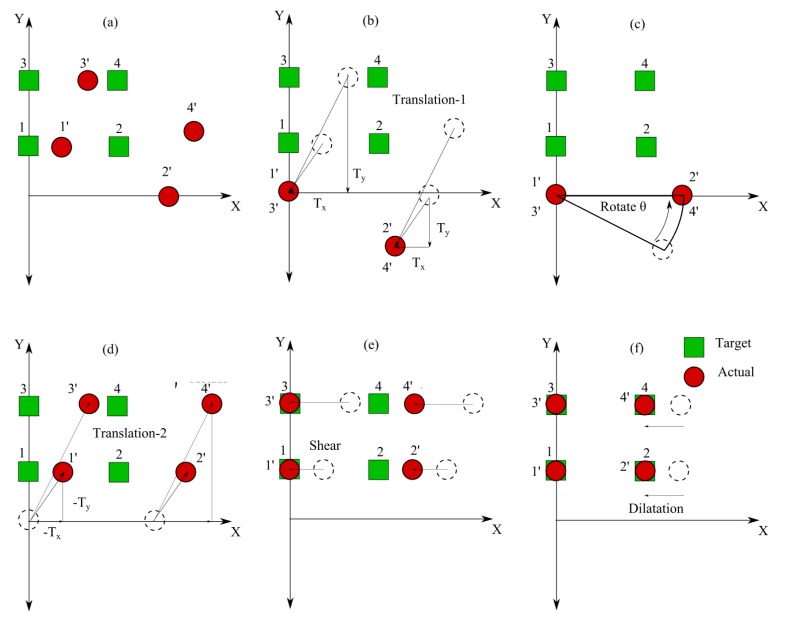
Geometry processes to build the eye gaze motion tracking system using the EOG. (**a**) Actual positions compared to the targets. (**b**) Translation of the reference pixel to the center of the coordinate (Translation-1). (**c**) Rotation of the actual pixel to the center of the coordinate. (**d**) Translation with opposite scale factor from the translation in (Translation-2). (b). (**e**) Shear. (**f**) Dilatation.

A homogeneous matrix of the affine transform, based on the basic affine transform in Appendix B, was proposed to match the actual pixels with the target pixels in this research to compensate the translation processes, which are not linear with other translation processes [17]. Each of the four areas had a homogeneous matrix. In this study, the matrices were built from the sequences of the five geometry processes. Figure 8 illustrates how these processes worked.

Figure 8a illustrates the positions of the four actual pixels before the transformation in comparison to the target points. The translation process in Figure 8b was fundamental since the actual pixel was rotated about a certain point along the reference line. The rates of translation were determined from the projection of points to the reference lines. A rotation after the first translation is shown in Figure 8c. After the rotation, a translation geometry process was applied again to the actual pixels. As demonstrated by Figure 8d, the translation directions were opposite from those of the first translation. Therefore, the values of the translation were also reversed from the first translation procedure since they were vector units. The shear process played a role to decrease the error based on the reference lines, as demonstrated by Figure 8e. Finally, the dilatation process puts the actual pixels at the same positions as the target positions, as shown by Figure 8f. Equations (7)–(11) calculate the homogeneous matrices for each process. The homogeneous matrix for all processes could be determined by using the sequence of matrix multiplications denoted in Equation (12). The relationship between the actual pixel (u,v) and desired pixel (u',v') was determined by Equation (13).

(7) $$\text{Translation}-1=\left[\begin{array}{ccc} 1 & 0 & T_{x}\\ 0 & 1 & T_{y}\\ 0 & 0 & 1 \end{array}\right]$$

(8) $$\text{Rotation}=\left[\begin{array}{ccc} \cos\theta & -\sin\theta & 0\\ \sin\theta & \cos\theta & 0\\ 0 & 0 & 1 \end{array}\right]$$

(9) $$\text{Translation}-2=\left[\begin{array}{ccc} 1 & 0 & -T_{x}\\ 0 & 1 & -T_{y}\\ 0 & 0 & 1 \end{array}\right]$$

(10) $$\text{Shear}=\left[\begin{array}{ccc} 1 & m_{1} & 0\\ m_{2} & 1 & 0\\ 0 & 0 & 1 \end{array}\right]$$

(11) $$\text{Dilatation}=\left[\begin{array}{ccc} s_{1} & 0 & 0\\ 0 & s_{2} & 0\\ 0 & 0 & 1 \end{array}\right]$$

(12) $$\begin{array}{c} \text{Homogeneous Matrix}=\left[\text{Dilatation}\right]\left[\text{Shear}\right]\left[\text{Translation}-2\right]\left[\text{Rotation}\right]\left[\text{Translation}-2\right]\\ =\left[\begin{array}{ccc} s_{1}\left(\cos\theta +m_{1}\sin\theta \right) & s_{1}\left(-\sin\theta +m_{1}\cos\theta \right) & a_{1}\\ s_{2}\left(\sin\theta +m_{2}\cos\theta \right) & s_{2}\left(\cos\theta +m_{2}\sin\theta \right) & a_{2}\\ 0 & 0 & 1 \end{array}\right]\\ a_{1}=s_{1}\left(\left(T_{x}\cos\theta -T_{y}\sin\theta -T_{x}\right)+m_{1}\left(T_{x}\sin\theta +T_{y}\cos\theta -T_{y}\right)\right)\\ a_{2}=s_{2}\left(\left(T_{x}\sin\theta -T_{y}\cos\theta -T_{y}\right)+m_{2}\left(T_{x}\cos\theta -T_{y}\sin\theta -T_{x}\right)\right) \end{array}$$

(13) $$\left[\begin{array}{c} {\text{u}}^{\prime }\\ {\text{v}}^{\prime }\\ 1 \end{array}\right]=\text{Homogeneous Matrix}\left[\begin{array}{c} u\\ v\\ 1 \end{array}\right]$$

### 3.4. Process of Robot Control Using EOG

**Figure 9.**
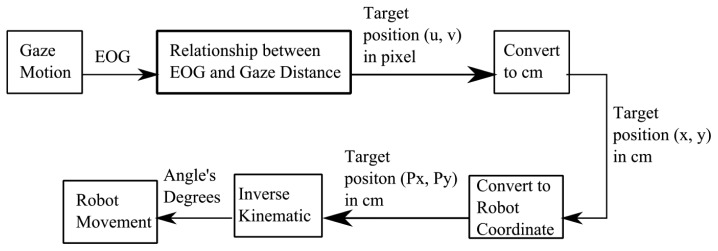
The process of robot control using EOG.

Figure 9 shows the overall process of controlling a robot using EOG. Gaze motions generated the EOG signals. Then, these signals were processed to get the target positions (u,v) of gaze motions based on the affine transformation in Equation (13) of actual pixels which is generated by linear relationship between EOG and gaze distance in Equation (3). Then, the pixel unita were converted to cm units by using Equation (5). The end-effector position (P_x_,P_y_) was calculated by Equation (4). Using inverse kinematic, the degrees of two angles were determined by Equations (A5) and (A6). These values were sent through serial communication to move the robot manipulator.

## 4. Result and Discussion

The tracking object system using the EOG was evaluated by three operators. The locations of the target points were exactly the same as those of the training targets, so the total number of target points was 24. Homogeneous matrices were built by the sequence of the five geometry processes. Two of the processes, which were rotation and dilatation, had rational numbers. On other hand, the translations and shear had dynamic variables. The values of the dynamic variables depended on the reference lines.

Table 3 provides the values of constant variables *a* and *b* for reference Equation (8) in the four areas. The result shows that the slopes of the reference lines followed some patterns. Areas 1 and 3 had positive slope (*a*) values, whereas Areas 2 and 4 had negative slope values. The highest positive gradient was 2.44 and the lowest negative gradient was −1.07. The average slopes were 1.03 in Area 1 and 1.85 in Area 3. On the other hand, the average slopes in Areas 2 and 4 were −0.20 and −0.52, respectively.

These gradients confirm that the three operators showed the same pattern for actual pixels rotating to the real target coordinates in all four areas. The EOG signals not only depended on the individual but also on the environmental condition. Therefore, fluctuations occurred for rational number *b*.

**Figure 10.**
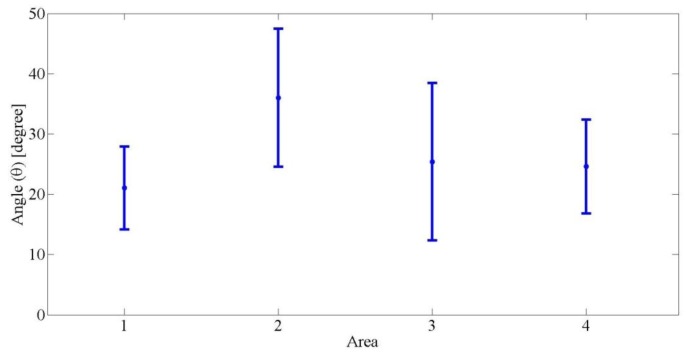
Average angle (θ) for the three operators for the rotation geometry process in the four areas.

For the rotation process, the angle varied for all areas. Figure 10 shows the average angles for the three operators. The maximum value of the angle was 47.70° and the minimum was 13.70°. The average angle in Area 1 was 21.03°. This value was not too different compared to Areas 3 and 4, which were 25.30° and 24.60°, respectively. On the other hand, it was 36.02° in Area 2 on average, and the average of the rotation angles was 26.76°.

**Figure 11.**
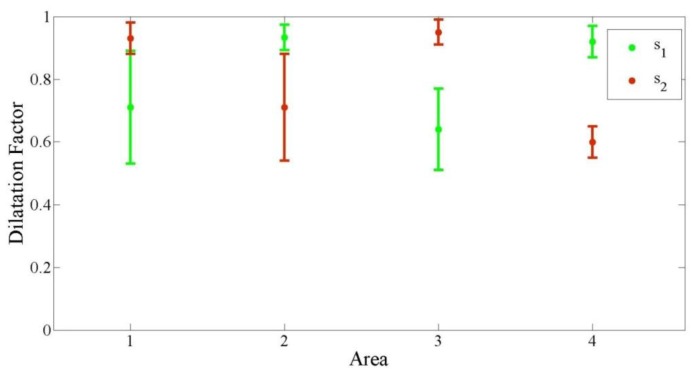
Average dilatation factor for horizontal (s_1_) and vertical (s_2_) for the three operators in the four areas.

Figure 11 shows the dilatation factor for the three operators in the four areas. The dilatation factors were classified into two types. The first was s_1_, which reconstructed the actual pixels in the horizontal direction (x-axis). The second was s_2_, which reconstructed the actual pixels in the vertical direction (y-axis). The result shows that the dilatation factor also had a pattern. For the horizontal geometry process, the average horizontal dilatation factors in Areas 1 and 3 were almost same, 0.71 and 0.63, respectively. These numbers were also the same for the vertical dilatation factor in Areas 2 and 4, which were 0.70 and 0.60. At a glance, the same values were also generated among Areas 2 and 4 in the horizontal direction and Areas 1 and 3 in the vertical direction. The average vertical dilatation factors for Areas 1 and 3 in the horizontal dilatation process were 0.93 and 0.94, whereas the average horizontal dilatation factors for Areas 2 and 4 were 0.93 and 0.92. This result shows that the actual pixels were always bigger than the target pixels. For Areas 1 and 3, the extension in the vertical direction had a bigger impact than that in the horizontal. But, Areas 2 and 4 had bigger expansion in the horizontal direction than in the vertical.

**Figure 12.**
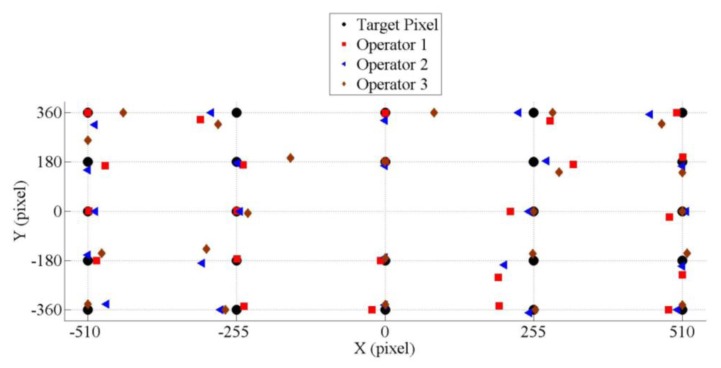
The average positions of actual pixels and 24 target pixels for the three operators.

The three operators tried to gaze at the target points. Figure 12 illustrates the actual pixel positions that the three operators gazed at while looking to the target points. The best performance of this system was when the operators gazed to the horizontal or vertical positions only. In this case, the average error in Area 1 was 18 ± 4 pixels, Area 2 was 12 ± 7, Area 3 was 11 ± 4 and Area 4 was 14 ± 9. Overall, the average error in the horizontal direction was approximately 11 ± 9 pixels and it was 7 ± 6 pixels in the vertical.

The pixel errors were converted to the angle of gaze motion to make it more general. The normal distance between the operators and the targets was 40 cm. The height of target area was 27 cm and the width of it was 32 cm. Number of pixels in horizontal was 1020 pixels and number of pixel in vertical was 720 pixels. Every pixel in the horizontal direction was equal to 0.033 cm and it was 0.0375 cm in the vertical direction. So, by using trigonometry, the average error angle in the horizontal direction was 0.86° ± 0.67° and 0.54° ± 0.34° in the vertical.

The tracking system using EOG was implemented to control a robot manipulator. Table 4 shows the angle degrees of two joints, *α* and *β*, from three operators who moved the end-effector to six target points. Three operators could use the system well. They controlled the robot and it successfully tracked the gaze motions by moving it to the target points.

## 5. Conclusions

This experiment was done by using a 40 cm target plate in front of an operator with limitation of the linear area to about 46° in horizontal and 38° in vertical gaze motions. Since there are many EOG recording techniques [14], electrode positions had to be first inspected in order to study the signal conditions before implementing a tracking system using EOG. The electrode positions could be different among the devices [18,19]. The skin condition is also an important factor to keep the signals stable. In this research, a low impedance and high conductivity gel was used as the surface between electrodes and skin. This system produces a stable EOG signal during the experiment as long as the head is in a fixed position.

This research improved the performance of tracking an object by using EOG signals. In other research [20], the relationship between eye movements and EOG was measured. Simulated EOG signals were identified by neural networks. This method reached a approximate accuracy of ±1.09°.

Another important issue in the rotation process was that the actual pixels were rotated to certain points that belonged to the reference lines. These lines were also useful for the translation process. The previous research [14] rotated the actual pixels to the center of the coordinate of the target pixel. The new technique proved that improvement was achieved in all areas of gaze motions.

One of the challenges of the affine transform method was obtaining a simpler method to determine the variables of the homogeneous matrices. Four homogeneous matrices were developed for the four areas, and each homogeneous matrix has the following seven variables that need to be determined: translation variables (T_x_,T_y_), rotation variable (θ), shear variables (m_1_,m_2_) and dilatation variables (s_1_,s_2_). This method was successfully to track the gaze motions not only in directions but also in distance that was indicated by the movement of the planar robot manipulator.

## Figures and Tables

**Table 1.** Combinations of EOG Signals.

	**Area 1**	**Area 2**	**Area 3**	**Area 4**
Ch1	Negative (−)	Positive (+)	Positive (+)	Negative (−)
Ch2	Negative (−)	Negative (−)	Positive (+)	Positive (+)

**Table 2.** Pixel locations of each training target.

**Area 1**			**Area 2**			**Area 3**			**Area 4**		

**Target**	**Pixel**		**Target**	**Pixel**		**Target**	**Pixel**		**Target**	**Pixel**	
			
	**x**	**y**		**x**	**y**		**x**	**y**		**x**	**y**
0,1	0	180	1,0	255	0	0, −1	0	−180	−1,0	−255	0
0,2	0	360	1,−1	255	−180	0, −2	0	−360	−1,1	−255	180
1,1	255	180	1,−2	255	−360	−1, −1	−255	−180	−1,2	−255	360
1,2	255	360	2,0	255	0	−1, −2	−255	−360	−2,0	−255	0
2,1	510	180	2, −1	510	−180	−2, −1	−510	−180	−2,1	−510	180
2,2	510	360	2, −2	510	−360	−2, −2	−510	−360	−2,2	−510	360

**Table 3.** Constant variables for reference equations.

**Area**	**Variable**	**Operator 1**	**Operator 2**	**Operator 3**
1	*a*	1.27	0.90	0.89
	*b*	6.99	−93.82	416.4

2	*a*	−0.30	−0.12	−0.14
	*b*	−155.94	68.44	11.99

3	*a*	2.08	2.44	1.00
	*b*	84.70	80.60	−241.55

4	*a*	−1.07	−0.41	−0.08
	*b*	−560.90	158.36	70.88

**Table 4.** Joint angles of robot manipulator for six target points from three operators.

**Targets**		**Operator 1**		**Operator 2**		**Operator 3**	
u	v	*α*°	*β*°	*α*°	*β*°	*α*°	*β*°
300	180	56	107	60	105	58	109
180	−50	64	119	64	120	64	120
−300	−150	96	103	99	99	98	97
−200	180	91	82	92	81	88	84
−510	−180	111	85	111	82	110	84
510	360	52	94	52	97	54	94

**Figure A1.**
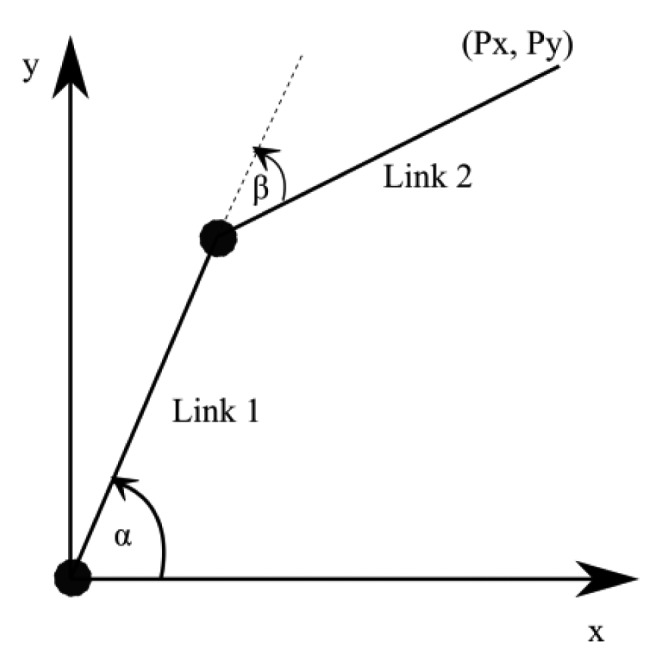
Base coordinates of robot manipulator.

## Appendix A.

This research used a 2-degree of freedom planar robot manipulator to indicate the gaze motions. The first link (Link 1) and the second link (Link 2) were 30 cm in length. The angle for joint 1 was *α* and angle for joint 2 was *β*. The end-effector position was (Px,Py). The coordinate of the robot was shown by Figure A1. The relationship between end-effector position and the joint angles was calculated by using *a tan* 2 function as shown by Equations (A1)–(A7).

(A1) Link1=Link2=Link

(A2) $$\cos\beta =\left(\frac{P^{2}x+P^{2}y-2\times {\text{link}}^{2}}{2\times {\text{Link}}^{2}}\right)$$

(A3) $$\cos\alpha =\left(\frac{\text{Link}\times \left(Px\times \left(1+\cos\beta \right)+Py\times \left(\sqrt{1-\cos^{2}\beta }\right)\right)}{P^{2}x+P^{2}y}\right)$$

(A4) $$\sin\beta =\sqrt{1-\left(\frac{P^{2}x+P^{2}y-2\times {\text{Link}}^{2}}{2\times {\text{Link}}^{2}}\right)}$$

(A5) $$\sin\alpha =\sqrt{1-{\left(\frac{\text{Link}\times \left(Px\times \left(1+\cos\beta \right)+Py\times \left(\sqrt{1-\cos^{2}\beta }\right)\right)}{P^{2}x+P^{2}y}\right)}^{2}}$$

(A6) β=atan2(cosβ,sinβ)

(A7) α=atan2(cosα,sinα)

## Appendix B.

In this research, affine transformation was used to find the 2-dimensional pixel coordinates of eye movements. Consider that a target is *A* (*u, v*) and the actual pixel produced by the EOG signal is *A*′ (*u*′, *v*′). In general, the affine transform constructs the relationship between *A* and *A*′, as shown in Equation (B1). This formula has the ability to provide geometry processes such as counterclockwise rotation in Equation (B2), clockwise rotation in Equation (B3), translation in Equation (B4), shear in Equation (B5) and dilatation in Equation (B6):

(B1) $$\left[\begin{array}{c} {\text{u}}^{\prime }\\ v \end{array}\right]=\left[\begin{array}{cc} c_{1} & c_{2}\\ c_{3} & c_{4} \end{array}\right]\left[\begin{array}{c} u\\ v \end{array}\right]+\left[\begin{array}{c} T_{u}\\ T_{v} \end{array}\right]$$

(B2) $$\begin{array}{c} \left[\begin{array}{c} u^{\prime }\\ v^{\prime } \end{array}\right]=\left[\begin{array}{cc} \cos\theta & -\sin\theta \\ \sin\theta & \cos\theta \end{array}\right]\left[\begin{array}{c} u\\ v \end{array}\right]\\ c_{1}=c_{4}=\cos\theta ,c_{2}=-\sin\theta ,c_{3}=\sin\theta ,T_{u}=T_{v}=0 \end{array}$$

(B3) $$\begin{array}{c} \left[\begin{array}{c} u^{\prime }\\ v^{\prime } \end{array}\right]=\left[\begin{array}{cc} \cos\theta & \sin\theta \\ \sin\theta & \cos\theta \end{array}\right]\left[\begin{array}{c} u\\ v \end{array}\right]\\ c_{1}=c_{4}=\cos\theta ,c_{2}=c_{3}=\sin\theta ,T_{u}=T_{v}=0 \end{array}$$

(B4) $$\begin{array}{c} \left[\begin{array}{c} u^{\prime }\\ v^{\prime } \end{array}\right]=\left[\begin{array}{cc} 1 & 0\\ 0 & 1 \end{array}\right]\left[\begin{array}{c} u\\ v \end{array}\right]+\left[\begin{array}{c} T_{u}\\ T_{v} \end{array}\right]\\ c_{1}=c_{4}=1,c_{2}=c_{3}=0\\ T_{u}=\text{Horizontal translation factor},T_{v}=\text{Vertical translation factor} \end{array}$$

(B5) $$\begin{array}{c} \left[\begin{array}{c} u^{\prime }\\ v^{\prime } \end{array}\right]=\left[\begin{array}{cc} 1 & m_{1}\\ m_{2} & 1 \end{array}\right]\left[\begin{array}{c} u\\ v \end{array}\right]\\ c_{1}=c_{4}=1,T_{u}=T_{v}=0\\ m_{1}=\text{Vertical shear factor},m_{2}=\text{Horizontal shear factor} \end{array}$$

(B6) $$\begin{array}{c} \left[\begin{array}{c} u^{\prime }\\ v^{\prime } \end{array}\right]=\left[\begin{array}{cc} s_{1} & 0\\ 0 & s_{2} \end{array}\right]\left[\begin{array}{c} u\\ v \end{array}\right]\\ c_{2}=c_{3}=T_{u}=T_{v}=0\\ s_{1}=\text{Horizontal scale factor},s_{2}=\text{Vertical scale factor} \end{array}$$
